# ﻿A new species of the genus *Hebius* (Squamata, Natricidae) from Yunnan, China

**DOI:** 10.3897/zookeys.1206.123841

**Published:** 2024-07-09

**Authors:** Yu-Hao Xu, Dian-Cheng Yang, Yan-An Gong, Kai-Chen Ouyang, Shi-Yang Weng, Jun-Dong Deng, Song Huang, Li-Fang Peng

**Affiliations:** 1 State Key Laboratory of Plateau Ecology and Agriculture, Qinghai University, Xining 810016, China Qinghai University Xining China; 2 Anhui Province Key Laboratory of the Conservation and Exploitation of Biological Resource, College of Life Sciences, Anhui Normal University, Wuhu 241000, China Anhui Normal University Wuhu China; 3 Tibet Plateau Institute of Biology, Lhasa 850008, China Tibet Plateau Institute of Biology lhasa China

**Keywords:** Cyt *b*, *Hebiuscitrinoventer* sp. nov., keelback snake, molecular systematics, Natricinae, taxonomy, Yingjiang County

## Abstract

A new species of the genus *Hebius* Thompson, 1913 is described from Yingjiang County, Dehong Dai and Jingpo Autonomous Prefecture, Yunnan Province, China, based on molecular and morphological evidence. It can be distinguished from its congeners by the following set of characters: (1) dorsal scale rows 19–17–17, feebly keeled; (2) ventrals 146–151; (3) nasal complete, nostril in the middle of the nasal; (4) supralabials 9, the fourth to sixth in contact with the eye; (5) infralabials 10–11, the first 5 touching the first pair of chin shields; (6) preoculars 2; (7) postoculars 3; (8) temporals 3, arranged in two rows (1+2); (9) maxillary teeth 31, the last 4 slightly enlarged, without diastema; (10) tail comparatively long, TAL/TL ratio 0.334 in the male; (11) dorsolateral series of irregular orange or ochre yellow blotches, extending from the neck to the posterior part of the tail; and (12) venter pale orange, tips of ventrals with subrectangular black blotches. All *Hebius* specimens were strongly recovered as monophyletic, in which *Hebiustaronensis* (Smith, 1940) and *Hebiusvenningi* (Wall, 1910) were monophyletic as sister to the Yingjiang County specimens. According to the *p*‐distance of cytochrome *b*, the new species differs from its congeners by 9.7–15.4%.

## ﻿Introduction

The natricine snake genus *Amphiesma* Duméril, Bibron & Duméril, 1854 long represented a genus of small- to medium-sized, semi-aquatic species, widely distributed from South to Southeast Asia (Guo et al. 2014; Zhou et al. 2019; David et al. 2021). Because of interspecific morphological similarities and lack of broader genetic and morphological sampling, the systematic conflict of this group at the genus and species levels long persists. Based on molecular characteristics, Guo et al. (2014) split the genus *Amphiesma* into three genera: *Amphiesma*; *Hebius* Thompson, 1913, which accounts for most of the species; and *Herpetoreas* Günther, 1860. Kizirian et al. (2018) found that the genera *Parahelicops* Bourret, 1934 and *Pararhabdophis* Bourret, 1934 were junior synonyms of *Hebius* which was also supported by Ren et al. (2018). The molecular data presented by Patel et al. (2023) revealed a new genus, *Sahyadriophis* Patel, Thackeray, Campbell & Mirza, 2023, in which *Hebiusbeddomei* (Günther, 1864) was included. In addition, the generic assignments of some species have also changed. For example, *Hebiusxenura* (Wall, 1907) and *Hebiuspealii* (Sclater, 1891) have been assigned to *Herpetoreas*, and *Hebiusmonticola* (Jerdon, 1853) has been assigned to *Amphiesma* (Kizirian et al. 2018; Ren et al. 2018; Das et al. 2020; Lalronunga et al. 2020; Ren et al. 2022; Patel et al. 2023).

Currently, there are 51 valid species in the genus *Hebius*, of which 26 are known to occur in China (Hauser et al. 2022; Li et al. 2022; Ma et al. 2023; Xu et al. 2023; Uetz et al. 2024). Furthermore, recent morphological and molecular phylogenetic analyses of *Hebius* have shown that the weak species delimitation within this genus may be due to underestimated diversity (Guo et al. 2014; Kizirian et al. 2018; Liu et al. 2018; Zhou et al. 2019; Hou et al. 2021).

**Figure 1.**
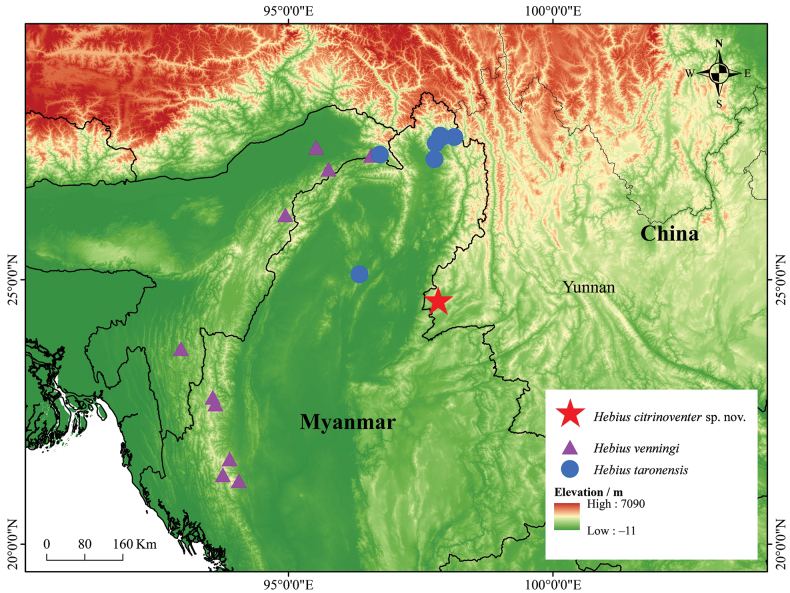
Distribution of selected species of the genus *Hebius*. Red star: *Hebiuscitrinoventer* sp. nov.; purple triangle: *H.venningi*; blue circle: *H.taronensis*.

Our morphological and molecular results support the presence of a new snake species, based on two specimens collected from Yingjiang County, Yunnan Province, China (Fig. 1) during field surveys in July 2023 and February 2024. The specimens could be identified as members of *Hebius* by having the following combination of morphological characters: (1) 2 supralabials in contact with nasal; (2) maxillary teeth in a continuous series, gradually larger posteriorly in the series or the last two teeth abruptly enlarged, the diastema before the distinctly enlarged posterior maxillary teeth absent; (3) internasals broad anteriorly, nostrils lateral; and (4) color pattern usually comprising a dorsolateral series of dark dots, forming two longitudinal stripes. However, these specimens could not be assigned to any known species (Guo et al. 2014; Ren et al. 2018). Furthermore, molecular analyses also revealed that the Yingjiang County specimens differed from those of other congeners.

## ﻿Material and methods

### ﻿Sampling

One drowned adult female and one road-killed subadult male specimens were collected from Yingjiang County, Dehong Dai and Jingpo Autonomous Prefecture, Yunnan Province, China. Sex was determined by tail dissection. Liver tissues were extracted and preserved in 95% ethanol. The specimens were preserved in 75% ethanol, and deposited in the Anhui Normal University Museum (ANU) and Qinghai University Museum (QHU). All sampling and procedures involving snake specimens were performed in accordance with the Wild Animals Protection Law of the People’s Republic of China and approved by the Institutional Ethics Committee of Anhui Normal University (protocol code AHNU-ET2021025) and Qinghai University (protocol code SL-2023028).

### ﻿Molecular phylogeny

Total genomic DNA was extracted from ethanol-preserved liver tissues using the QIAamp DNA Mini Kit (QIAGEN, Changsheng Biotechnology Co. Ltd., Changchun, China). We amplified the fragments of cytochrome *b* (Cyt *b*) by the Polymerase Chain Reaction (PCR), using the primers L14910 (5’-GAC CTG TGA TMT GAA AAC CAY CGT TGT-3’) and H16064 (5’-CTT TGG TTT ACA AGA ACA ATG CTT TA-3’) (Burbrink et al. 2000). The PCR products were sequenced by Shanghai Map Biotech Co., Ltd. (Shanghai, China). The raw sequences were stitched using SeqMan in the DNASTAR software package (Burland 2000) and the newly generated sequences were submitted to GenBank (accession numbers: PP472750, ANU20230016; PP429724, QHU2024005).

For phylogenetic analysis, 56 sequences were selected (Table 1), among which 54 (No. 3–56) were obtained from the National Center for Biotechnology Information (NCBI), including 51 sequences from 31 *Hebius* species and three outgroups: *Trachischiummonticola* (Cantor, 1839), *Herpetoreasplatyceps* (Blyth, 1854), and *Herpetoreasburbrinki* Guo, Zhu, Liu, Zhang, Li, Huang & Pyron, 2014, and aligned using MEGA X software (Kumar et al. 2018). Tree inference was performed in IQ-TREE v. 1.6.12 (Nguyen et al. 2015) under the maximum likelihood (ML) model, and 5000 replicates of ultrafast bootstrap were used to estimate the Ultrafast Bootstrap Approximation (UFB) node support. The SH-like approximate likelihood ratio test (SH-aLRT) was conducted with 1000 replicates. In addition, we calculated the uncorrected pairwise distances (*p*-distances) using the MEGA X software (Kumar et al. 2018).

**Table 1.** GenBank accession numbers, localities, and voucher information for all specimens used in this study.

ID	Species name	Locality	Voucher	Cyt *b*	Reference
1	*Hebiuscitrinoventer* sp. nov.	Yingjiang, Yunnan, China	ANU20230016	PP472750	This study
2	*H.citrinoventer* sp. nov.	Yingjiang, Yunnan, China	QHU2024005	PP429724	This study
3	*H.andreae*	Khammouane, Laos	VNUF R.2017.25	MK253674	Ziegler et al. 2019
4	*H.annamensis*	Laos	FMNH 258637	OK315812	Deepak et al. 2022
5	*H.atemporalis*	Vietnam	ZMMU NAP-07877	OK315813	Deepak et al. 2022
6	*H.atemporalis*	Guangdong, China	GP 1626	KJ685680	Guo et al. 2014
7	*H.bitaeniatus* 1	Guangxi, China	GP 1940	KJ685688	Guo et al. 2014
8	*H.bitaeniatus* 2	Thailand	AUP-00062	OK315816	Deepak et al. 2022
9	*H.boulengeri*	Fujian, China	GP 2433	KJ685699	Guo et al. 2014
10	*H.boulengeri*	Guangdong, China	GP 1789	KJ685684	Guo et al. 2014
11	*H.chapaensis*	Lao Cai, Vietnam	VNMN 06102	MH778702	Ren et al. 2018
12	*H.chapaensis*	Lao Cai, Vietnam	VNMN 06103	MH778700	Ren et al. 2018
13	*H.clerki*	Pianma, Lushui, Yunnan, China	KIZ037714	MZ570478	Hou et al. 2021
14	*H.concelarus*	Miyakojimashi, Ryukyu, Japan	KUZ R18555	AB989258	Takuma and Toda 2016
15	*H.concelarus*	Miyakojimashi, Ryukyu, Japan	KUZ R20253	AB989268	Takuma and Toda 2016
16	*H.craspedogaster*	Guizhou, China	GP 1240	KJ685672	Guo et al. 2014
17	*H.deschauenseei*	Thailand	AUP-00182	OK315827	Deepak et al. 2022
18	*H.igneus*	Ha Giang, Vietnam	AMNH 148575	KJ685665	Guo et al. 2014
19	*H.ishigakiensis*	Ishigakishi, Ryukyu, Japan	KUZ R19251	AB989282	Takuma and Toda 2016
20	*H.ishigakiensis*	Taketomityo, Ryukyu, Japan	KUZ R33043	AB989292	Takuma and Toda 2016
21	*H.jingdongensis*	Jingdong, Yunnan, China	CIB 119044	OR285310	Ma et al. 2023
22	*H.johannis*	Yunnan, China	KIZ014484	MZ570479	Hou et al. 2021
23	*H.khasiensis*	Kachin state, Myanmar	CAS 221504	KJ685668	Guo et al. 2014
24	*H.khasiensis*	Kachin state, Myanmar	CAS 221525	KJ685669	Guo et al. 2014
25	*H.maximus*	Sichuan, China	GP 864	KJ685706	Guo et al. 2014
26	*H.maximus*	Sichuan, China	GP 2382	KJ685696	Guo et al. 2014
27	*H.metusia*	Shimian, Sichuan, China	KIZ05178	MZ570480	Hou et al. 2021
28	*H.metusia*	Sichuan, China	GP 871	KJ685707	Guo et al. 2014
29	*H.modestus*	Yunnan, China	CAS 234262	KJ685671	Guo et al. 2014
30	*H.modestus*	Diantan, Tengchong, Yunnan, China	KIZ037715	MZ570481	Hou et al. 2021
31	*H.octolineatus*	Kunming, Yunnan, China	KIZ026445	MZ570484	Hou et al. 2021
32	*H.octolineatus*	Kunming, Yunnan, China	KIZ03204	MZ570483	Hou et al. 2021
33	*H.optatus*	Guizhou, China	GP 1885	KJ685687	Guo et al. 2014
34	H.cf.optatus	Vinh Phuc, Vietnam	AMNH 147155	KJ685662	Guo et al. 2014
35	*H.popei*	Hainan, China	GP 2169	KJ685692	Guo et al. 2014
36	*H.popei*	Guizhou, China	GP 2386	KJ685697	Guo et al. 2014
37	*H.pryeri*	Tokunoshimacho, Ryukyu, Japan	KUZ R34044	AB989124	Takuma and Toda 2016
38	*H.pryeri*	Ryukyu, Japan	KUZ R34062	AB989126	Takuma and Toda 2016
39	*H.sangzhiensis*	Hunan, China	SYNU08070350	MK340763	Zhou et al. 2019
40	*H.sauteri*	Taiwan, China	GP 2549	KJ685701	Guo et al. 2014
41	*H.sauteri*	Guangdong, China	CIB 118516	OP937178	Li et al. 2022
42	*H.septemlineatus*	Diantan, Tengchong, Yunnan, China	KIZ037706	MZ570485	Hou et al. 2021
43	*H.septemlineatus*	Zizhi, Tengchong, Yunnan, China	KIZ037720	MZ570486	Hou et al. 2021
44	*H.taronensis*	Myanmar	GP 1618	KJ685679	Guo et al. 2014
45	*H.taronensis*	Myanmar	CAS 224426	OK315828	Deepak et al. 2022
46	*H.venningi*	KaChin state, Myanmar	CAS 233206	KJ685670	Guo et al. 2014
47	*H.vibakari*	Liaoning, China	GP 1345	KJ685676	Guo et al. 2014
48	*H.vibakari*	Heilongjiang, China	GP 1352	KJ685677	Guo et al. 2014
49	*H.weixiensis*	Weixi, Yunnan, China	KIZ035740	MZ570488	Hou et al. 2021
50	*H.weixiensis*	Lijiang, Yunnan, China	HSR19088	OQ085074	Xu et al. 2023
51	*H.yanbianensis*	Yanbian, Sichuan, China	GP 4006	MH532291	Liu et al. 2018
52	*H.yanbianensis*	Binchuan, Yunnan, China	CIB5334220120	OR215499	Ma et al. 2023
53	*H.youjiangensis*	Baise, Guangxi, China	ANU20220010	OQ085073	Xu et al. 2023
**Out group**					
54	*Herpetoreasburbrinki*	Tibet, China	YBU 071128	GQ281781	Guo et al. 2014
55	*H.platyceps*	Tibet, China	GP 2096	KJ685690	Guo et al. 2014
56	*Trachischiummonticola*	Tibet, China	GP 1487	JQ687435	Guo et al. 2014

### ﻿Morphological examination

Morphological characters were described for the newly collected specimen and compared with other key references (Günther 1875; Wall 1910; Wall 1925; Bourret 1934; Taylor 1934; Gressitt 1937; Smith 1940; Dowling 1951a, 1951b; David and Das 2003; Zhao 2006; David and Vogel 2010; Guo et al. 2014; Liu et al. 2018; Ren et al. 2018; Purkayastha and David 2019; Zhou et al. 2019; Ziegler et al. 2019; David et al. 2021; Huang 2021; Hou et al. 2021; Hauser et al. 2022; Li et al. 2022; Ma et al. 2023; Xu et al. 2023). The measurements and scale counts followed those of Dowling (1951a, 1951b), Zhao (2006), and Huang (2021). A ruler with 1 mm accuracy was used to measure the snout-vent length (**SVL**), measured from the tip of the snout to the anterior edge of the vent ;tail length (**TAL**), measured from the anterior edge of the vent to the tip of the tail ; and total length (**TL**), defined as the sum of the SVL and TAL. All other measurement characteristics were measured to the nearest 0.01 mm using digital calipers ; head length (**HL**), measured from the tip of the snout to the posterior margin of the mandible : head width (**HW**), measured from the widest part of the head in dorsal view ; and eye diameter (**ED**), measured from the most anterior corner of the eye to the most posterior corner. Scalation features and their abbreviations are as follows : supralabials (**SL**) ; infralabials (**IL**) ; loreals (**LOR**) ; preoculars (**PRO**) ; postoculars (**PO**); Chin : infralabials touching the first pair of chin shields (**IFL**-1^st^ Chin) ; temporals (**TEMP**) ; supraoculars (**SPO**); and three dorsal scale row (**DSR**) counts: 1) counting from one head length behind the head, 2) at midbody (namely at SVL/2), and 3) at one head length before the vent ; ventral scales (**VS**) ; cloacal plate (**CP**); and subcaudal (**SC**). In addition, we also examined the number of maxillary teeth (**MT**).

## ﻿Results

### ﻿Phylogenetic relationships

**Figure 2.**
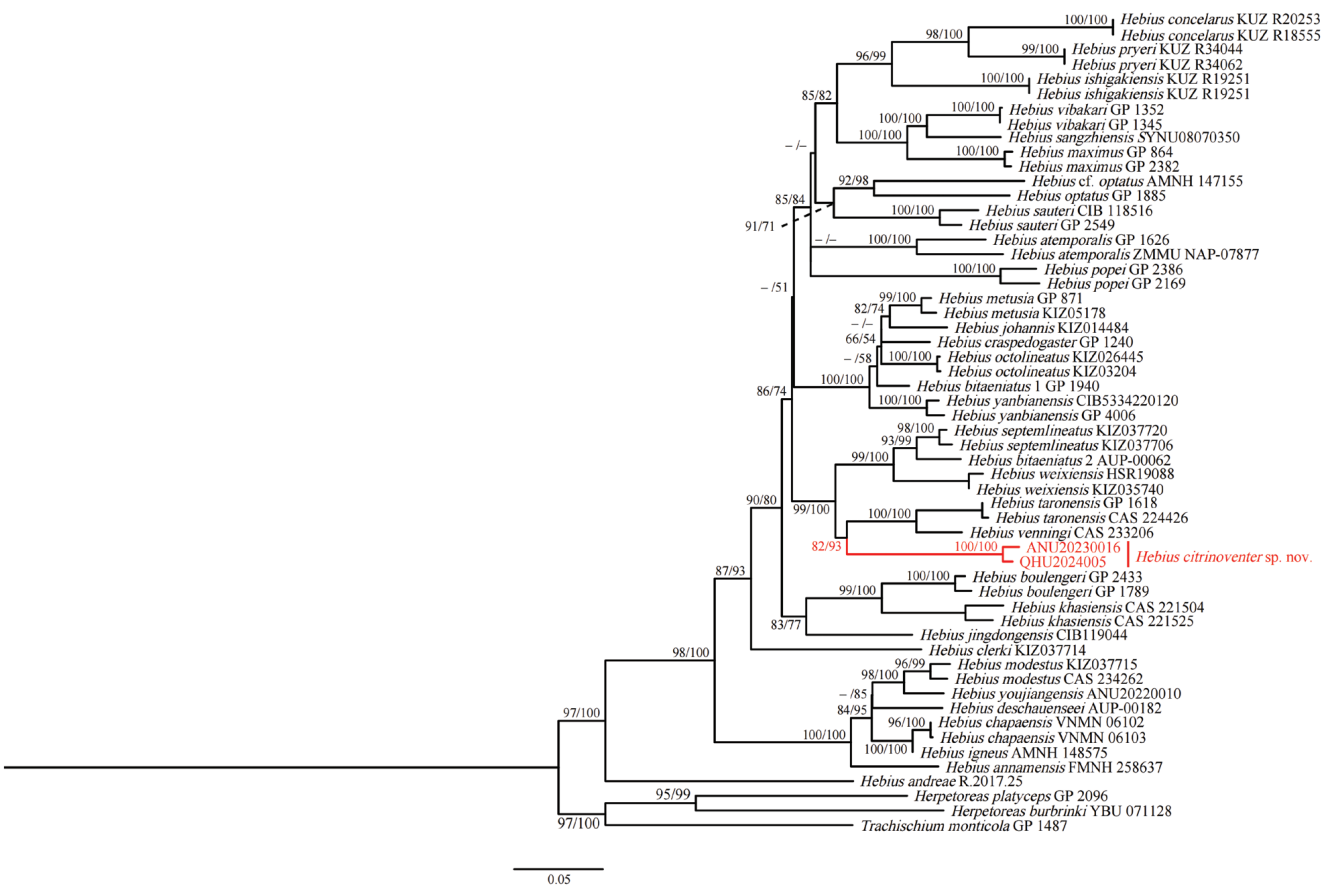
Maximum likelihood tree of the genus *Hebius* inferred from Cyt *b*. The nodes supporting values on branches are presented as SH-like approximate likelihood ratio test (SH) / Ultrafast Bootstrap Approximation (UFB); ones under 50% are omitted. Tips for the new species in the present study are shown in red.

The ML tree was reconstructed from a fragment of the mitochondrial Cyt *b* gene (Fig. 2). Due to the poor quality at both ends of the newly generated sequences, we cut the sequences to obtain a final length of 725 base pairs (bp). All *Hebius* specimens clustered into one monophyletic group with strong supports (SH 97 / UFB 100). *Hebiustaronensis* (Smith, 1940) and *H.venningi* (Wall, 1910) were grouped together (SH 100 / UFB 100), forming a sister clade to specimens from Yingjiang County (SH 82 / UFB 93), then clustering with *H.septemlineatus* (Schmidt, 1925), *H.weixiensis* Hou, Yuan, Wei, Zhao, Liu, Wu, Shen, Chen, Guo & Che, 2021, and the specimen AUP-00062 that was once identified as *H.bitaeniatus* (Wall, 1925). Regarding *p*-distance, the new species differed from its congeners by 9.7% (with *H.venningi*) to 15.4% (with *H.popei* (Schmidt, 1925)) (Table 2), which strongly suggests that the newly collected specimens have distinct genetic differentiation from their congeners. Moreover, morphological data supported the recognition of specimens from Yingjiang County as distinct from all other described species of *Hebius*. Thus, we describe the unnamed specimens as a new species.

**Table 2.** Uncorrected *p*-distances (%) among the *Hebius* species based on partial mitochondrial Cyt *b* gene.

ID	Species	1–2	3	4	5–6	7	8	9–10	11–12	13	14–15	16	17	18	19–20	21	22	23–24	25–26	27–28	29–30	31–32	33	34	35–36	37–38	39	40–41	42–43	44–45	46	47–48	49–50	51–52
**1–2**	*H.citrinoventer* sp. nov.	1.3																																
**3**	*H.andreae*	16.5–16.9	–																															
**4**	*H.annamensis*	14.2–14.5	12.7	–																														
**5–6**	*H.atemporalis*	12.5–13.7	14.2–15.2	12.5–12.7	6.1																													
**7**	*H.bitaeniatus 2*	10.0	15.9	12.7	10.7–11.0	–																												
**8**	*H. bitaeniatus1*	11.0–11.4	13.7	10.9	9.4–10.5	10.2	–																											
**9– 10**	*H.boulengeri*	13.7–14.5	16.2–16.4	12.2–12.4	11.7–12.2	10.4–10.7	10.4–10.5	1.2																										
**11–12**	*H.chapaensis*	12.9–13.2	12.7	5.7	11.0–11.4	12.2	10.5	12.0–12.2	0																									
**13**	*H.clerki*	13.7–14.0	15.5	12.2	10.4–12.2	12.7	11.5	10.5–10.7	11.0	–																								
**14–15**	*H.concelarum*	14.7–15.0	18.5	14.5	14.0–14.7	13.9	12.9	13.4–13.9	14.0	15.0	0																							
**16**	*H.craspedogaster*	11.4–11.7	14.7	11.0	9.4–11.2	11.5	3.5	11.2–11.4	10.9	12.0	14.4	–																						
**17**	*H.deschauenseei*	12.9–13.0	13.4	7.9	12.2–12.5	13.4	11.0	13.2–13.4	6.0	12.4	14.7	11.5	–																					
**18**	*H.igneus*	13.2–13.4	12.5	5.7	11.4–11.5	12.5	10.9	12.9–13.0	.9	11.5	14.5	11.2	5.7	–																				
**19–20**	*H.ishigakiensis*	12.9–13.2	17.0	12.4	12.0–12.2	12.4	12.2	11.9–12.0	12.5	11.9	12.5	13.2	13.5	12.9	0																			
**21**	*H.jingdongensis*	11.2–11.5	13.0	10.9	9.9–10.2	10.5	7.4	9.2–9.7	10.5	11.4	13.9	8.5	10.5	10.5	12.2	–																		
**22**	*H.johannis*	10.4–10.7	14.2	9.7	10.4–10.5	11.0	4.8	10.5–10.7	9.7	11.4	12.9	5.3	11.5	10.2	12.9	9.9	–																	
**23–24**	*H.khasiensis*	14.9–15.2	15.7–16.5	12.4–12.9	11.4–12.7	12.2–12.4	10.7–11.2	7.5–8.0	12.0–12.2	12.2–12.4	14.0–14.4	11.2–11.5	13.0	12.7–13.0	12.9–13.4	9.5–10.7	11.7	2.8																
**25–26**	*H.maximus*	13.9–14.4	13.0–13.2	12.7–13.4	11.2–13.2	12.0–12.2	10.4–10.7	13.2–13.7	11.9	13.0–13.4	14.5–14.9	11.0–11.4	11.7–11.9	11.9–12.0	12.0–12.7	11.9–12.2	12.5–12.7	13.7–14.4	0.7															
**27–28**	*H.metusia*	10.9–11.4	13.5–13.7	10.2–10.7	9.7–9.9	9.7–10.0	2.8–3.5	10.5–11.2	10.2	11.5–11.7	13.2–16.0	4.7	10.5–10.7	10.4–10.5	12.5–12.7	7.5–8.4	4.5–4.8	10.9–11.4	10.4–10.9	1.0														
**29–30**	*H.modestus*	14.2–15.2	13.4–14.2	6.7–7.0	12.0–13.5	13.4–13.7	12.5–12.9	13.5–14.2	5.0–5.3	12.2–12.9	15.7	13.0–13.2	6.2–6.8	4.8	12.4–12.7	11.7–12.0	12.5	13.9–14.2	12.5–13.2	12.4–12.9	2.3													
**31–32**	*H.octolineatus*	11.2–11.5	12.7–12.9	10.5–10.7	9.7–11.0	11.4	4.2–4.3	10.0–10.7	10.5–10.7	11.9–12.0	12.5–13.0	4.7–4.8	11.0–11.4	11.0	12.0–12.2	8.2–8.4	5.2–5.3	10.7–11.9	10.5–10.9	4.7–4.8	12.2–12.7	0.5												
**33**	H.cf.optatus	12.9	15.9	13.5	11.9–12.2	11.5	10.0	10.7–10.9	12.5	12.2	13.5	11.0	12.7	12.5	11.7	11.2	10.5	13.5	10.9–11.0	10.9–11.0	13.9–14.0	10.4	–											
**34**	*H.optatus*	12.7	17.0	13.0	11.5–11.7	11.2	10.4	12.5–12.7	11.2	13.2	13.4	11.0	12.9	11.9	11.7	11.7	11.4	13.4	10.0–10.4	10.0–10.5	13.7–14.7	10.9–11.4	9.9	–										
**35–36**	*H.popei*	14.2–15.4	15.9–16.5	13.2–13.5	11.0–12.9	13.0–13.4	10.7–11.4	13.5–13.9	13.2–13.5	12.7	15.4–15.7	11.5–12.5	13.2–13.5	13.5–13.9	12.4–13.4	11.4–11.7	12.4–12.5	12.9–14.0	11.2–12.2	11.2–11.9	13.0–13.9	11.9–12.0	11.4–13.2	10.7–11.2	4.0									
**37–38**	*H.pryeri*	14.4–14.7	18.2	14.7	13.5–13.9	13.5	12.5	12.9–13.0	14.5	13.7	9.0	14.2	15.0	15.0	11.5	13.7	13.4	14.7	12.9–13.0	12.7–12.9	15.4–16.7	12.4–12.5	14.0	13.5	14.4–14.5	0								
**39**	*H.sangzhiensis*	13.0–13.4	14.2	13.0	11.5–12.5	11.5	9.0	12.4–12.5	11.9	13.0	11.7	11.4	12.0	12.2	11.9	11.5	11.0	12.5–13.0	6.7–7.0	10.7–11.0	12.5–12.9	9.9–10.0	12.0	12.0	11.4	11.5	–							
**40–41**	*H.sauteri*	12.0–13.0	14.5–15.5	13.4–13.7	10.9–13.2	11.9–12.4	10.4–11.0	11.9–13.2	12.2–12.5	12.0	14.0	10.4–11.5	12.7–12.9	12.7–12.9	11.4–11.5	9.7–10.7	11.7	11.9–13.0	11.2–12.0	10.4–10.7	13.9–14.2	9.7–10.9	11.0–11.7	10.4–10.7	11.2–12.0	13.5–13.9	12.4–13.0	2.3						
**42–43**	*H.septemlineatus*	10.0–10.2	15.2–15.5	12.2–12.4	11.0–11.7	4.2	10.0–10.2	10.5–11.4	12.0–12.2	12.4–12.7	13.4–13.5	10.5–10.7	12.4–12.7	12.4–12.7	11.7–12.5	9.5–9.7	10.2–10.7	11.7–12.5	11.0–11.5	9.7–9.9	13.0–14.2	10.0–10.2	12.0–12.4	11.4–11.5	12.5–13.7	13.0	10.4–10.7	10.7–11.5	1.0					
**44–45**	*H.taronensis*	10.7–11.2	14.4–14.5	12.2–12.5	12.2–13.5	10.5–11.0	10.7–11.2	12.7–13.4	11.4–11.7	13.0–13.2	14.4–14.9	10.5–11.0	11.9–12.2	11.7–12.0	12.7–12.9	10.7–11.2	11.0–11.5	13.2–14.0	11.7–12.2	10.0–10.9	12.4–13.0	10.0–10.5	12.4–12.9	12.5–13.0	10.9–11.5	13.0–13.4	10.9–11.4	12.5–13.5	9.7–10.2	0.5				
**46**	*H.venningi*	9.7–10.0	13.2	11.4	11.0–11.2	8.7	9.0	12.7–12.9	10.2	12.5	13.9	10.5	11.7	10.5	12.2	10.2	9.4	13.0–13.4	12.4–12.7	9.4–9.7	11.4–11.7	9.5–9.9	11.5	12.0	11.4	12.7	10.9	11.7–12.4	8.5–8.8	5.8–6.0	–			
**47–48**	*H.vibakari*	12.4–12.7	14.4	13.0	10.2–12.5	11.7	9.7	12.7–12.9	11.4	11.7	13.4	11.0	11.9	11.7	12.0	10.0	11.0	12.5	7.9–8.2	10.4–10.5	12.9–13.7	10.4–10.5	12.9	10.4	12.5	13.0	7.2	12.0–12.4	10.2–10.7	11.5–12.0	11.2	0		
**49–50**	*H.weixiensis*	11.0–11.5	14.9–15.4	12.2	11.4–12.2	5.7–5.8	9.9–10.2	10.9–11.5	11.5–11.9	12.2–12.7	12.9–13.0	10.2–10.5	12.5–12.9	11.9–12.2	12.2–12.4	9.7–10.2	10.5–10.9	12.2–13.0	11.4–11.9	9.9–10.2	12.9–13.5	10.7–11.4	11.7–12.2	10.9–11.0	13.4–13.7	13.4–13.9	10.4–10.9	11.5–12.2	5.0–5.7	9.8–10.9	8.3–8.5	10.4–10.5	0.5	
**51–52**	*H.yanbianensis*	11.9–12.2	13.7–14.2	11.7–11.9	10.0–10.7	10.7–11.2	3.7–4.2	10.7–11.4	11.0–11.5	12.4–12.7	13.0–13.4	4.8–5.3	11.5–11.7	11.5–11.7	12.4–13.5	9.0–9.5	6.3–6.5	11.2–12.5	11.2–11.4	4.5–4.8	13.0–14.0	5.5–6.0	12.2–12.4	11.5	11.7–12.4	12.5–13.5	10.0–10.5	11.2–12.2	9.5–10.4	10.7–11.4	9.8–10.4	10.7–10.9	9.5–10.4	1.8
**53**	*H.youjiangensis*	14.0–14.2	13.9	6.7	11.7–12.7	13.4	11.5	13.2–13.4	5.2	11.5	15.4	12.2	5.5	4.7	13.0	10.2	12.0	13.2–13.9	12.7–12.9	11.2–11.4	3.7	11.5–11.9	13.5	14.0	13.5	15.4	12.2	12.9–13.2	13.0–13.4	12.4–12.7	12.0	12.5	12.9–13.2	12.7–13.0

### ﻿Taxonomic account

#### ﻿Natricinae Bonaparte, 1838

***Hebius* Thompson, 1913**

##### ﻿ Hebius citrinoventer

Taxon classificationAnimaliaSquamataNatricidae

Xu, Yang, Ouyang, Huang & Peng sp. nov.

BB0AF325-90A6-5ACE-ADEE-CCC0F3B7E1A8

https://zoobank.org/EFBD9EA8-897A-46F4-91AD-12E03C26E7F5

Figs 3 , 4 , 5

###### Material examined.

**Figure 3.**
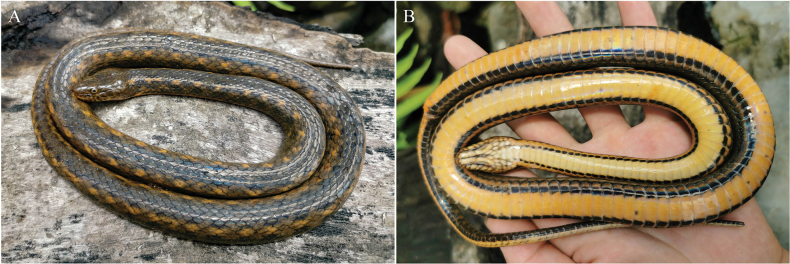
Fresh specimen of the holotype (ANU20230016) of *Hebiuscitrinoventer* sp. nov.: dorsal (**A**), and ventral views (**B**). Photos by Kai-Chen Ouyang. Scale bars are not shown.

**Figure 4.**
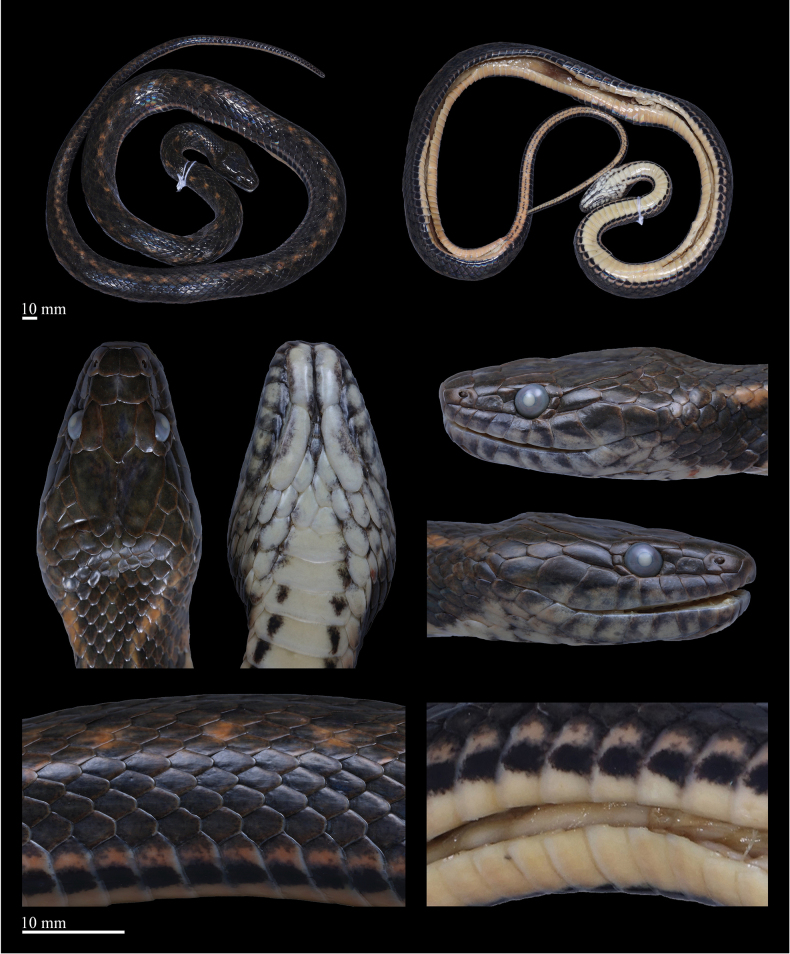
Preserved specimen of the holotype (ANU20230016) of *Hebiuscitrinoventer* sp. nov. Photos by Yu-Hao Xu. Scale bars: 10 mm.

**Figure 5.**
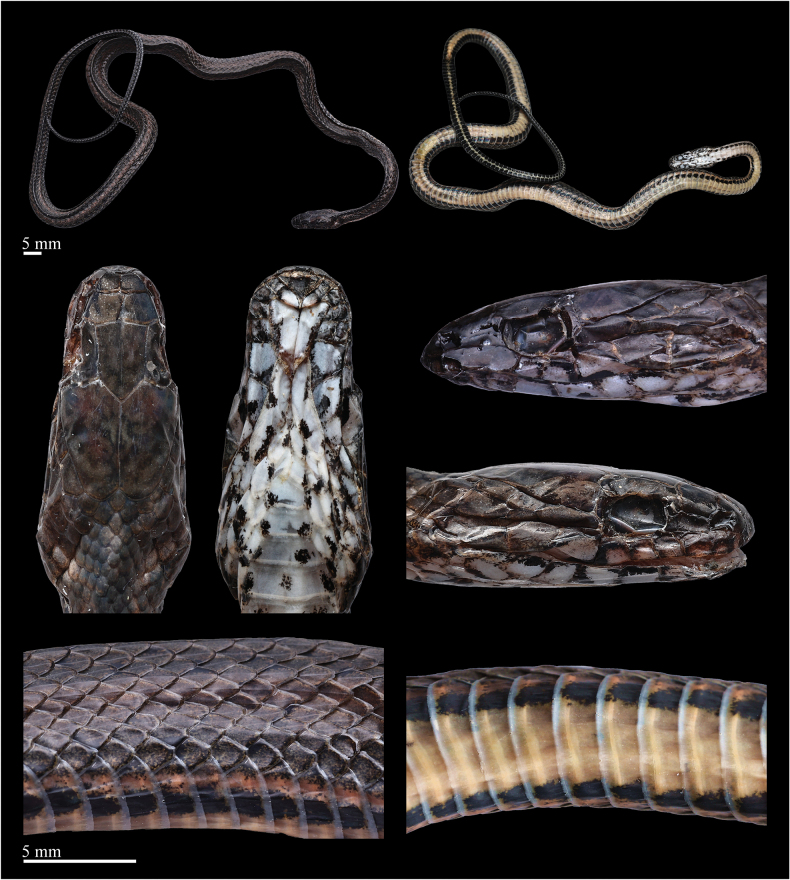
Preserved specimen of the paratype (QHU2024005) of *Hebiuscitrinoventer* sp. nov. Photos by Yu-Hao Xu. Scale bars: 5 mm.

***Holotype*.** ANU20230016 (field number: HSR23030, Figs 3, 4), an adult female, collected by Diancheng Yang and Jundong Deng on July 18, 2023, in Tongbiguan Town, Yingjiang County, Dehong Dai and Jingpo Autonomous Prefecture, Yunnan Province, China (24°36′30.60″N, 97°39′27.00″E, 1300 m a.s.l.). ***Paratype*.** QHU2024005 (field number: LFR2024007, Fig. 5), a subadult male, had been crushed to death on the side of the road, collected by Kaichen Ouyang and Lifang Peng on February 5, 2024, in Tongbiguan Town, Yingjiang County, Dehong Dai and Jingpo Autonomous Prefecture, Yunnan Province, China (24°36′03.60″N, 97°39′05.76″E, 1300 m a.s.l.).

###### Etymology.

The specific name *citrinoventer* comprises the Latin words “*citrinus*” (yellowish-orange or orange) and “*venter*” (the belly or underside), based on the pale orange venter of the new species. According to its type locality Yingjiang County, Yunnan Province, China, the name we suggest is Yíng Jiāng Fù Liàn Shé (盈江腹链蛇) in Chinese and Yingjiang Keelback Snake in English.

###### Diagnosis.

*Hebiuscitrinoventer* sp. nov. can be distinguished from its congeners by the following set of characters: (1) DSR 19–17–17, feebly keeled; (2) ventrals 146–151; (3) nasal complete, nostril in the middle of the nasal; (4) supralabials 9, the fourth to sixth in contact with the eye; (5) infralabials 10–11, the first 5 touching the first pair of chin shields; (6) preoculars 2; (7) postoculars 3; (8) temporals 3, arranged in two rows (1+2); (9) maxillary teeth 31, the last 4 slightly enlarged, without diastema; (10) tail comparatively long, TAL/TL ratio 0.334 in male; (11) dorsolateral series of irregular orange or ochre yellow blotches, extending from the neck to the posterior part of the tail; and (12) venter pale orange, tips of ventrals with subrectangular black blotches.

###### Comparisons.

**Figure 6.**
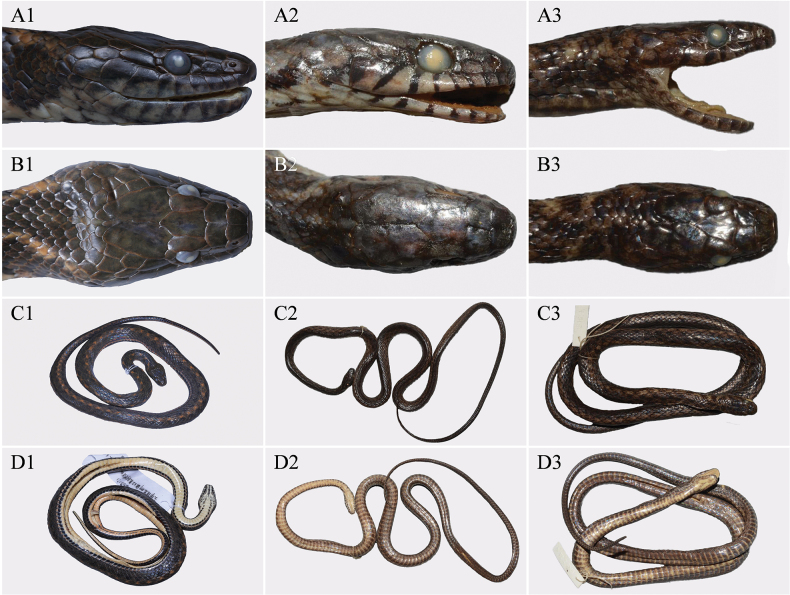
Comparisons of the lateral head (**A**), dorsal head (**B**), dorsal overview (**C**), and ventral overview (**D**) among the holotypes of *H.citrinoventer* sp. nov. (ANU20230016; **A1–D1**), *H.venningi* (BMNH 1946.1.21.86; **A2–D2**), and *H.taronensis* (BMNH 1946.1.13.55; **A3–D3**). Photos of *H.venningi* and *H.taronensis* were obtained from David et al. (2021), remaining photos by Yu-Hao Xu. Scale bars are not shown.

In many characters, *Hebiuscitrinoventer* sp. nov. is similar to *H.venningi* (Wall, 1910) and *H.taronensis* (Smith, 1940). However, the new species can be distinguished from *H.venningi* by (1) 19 DSR on the anterior part of the body (vs. 17), (2) VS 146–151 (vs. 155–172), (3) TEMP 1+2 (vs. 1 or 1+1); (4) maxillary teeth 31 (vs. 28–30), (5) venter pale orange, tips of ventrals with subrectangular black blotches (vs. venter pink or bright coral red, sometimes yellow). It can be distinguished from *H.taronensis* by (1) 17 DSR at midbody (vs. 19); (2) VS 146–151 (vs. 158–176); (3) TEMP 1+2 (vs. 1 or 1+1); (4) SC 113 (vs. 92–104); (5) tail comparatively longer, TAL/TL ratio 0.334 (vs. TAL/TL ratio 0.254–0.288); and (6) venter pale orange, tips of ventrals with subrectangular black blotches (vs. pale areas of the venter are yellowish-ochre or yellowish-brown). For more detailed information and visual comparisons, please refer to Table 3 and Fig. 6.

**Table 3.** Comparisons of main morphological characters between *H.citrinoventer* sp. nov., *H.taronensis* and *H.venningi*. Abbreviations as per Material and methods.

Species	TAL/TL	SL	IL	TEMP	DSR	VEN	SC	MT	Venter background coloration
*H.citrinoventer* sp. nov.	0.334	9	10–11	1+2	19–17–17	146–151	113	31	pale orange
*H.taronensis*	0.254–0.288	9 (rarely 8)	10	1 or 1+1	19–19–17	158–176	92–104	28–32	yellowish-ochre or yellowish-brown
*H.venningi*	0.295–0.347	9	9–10	1+1	17–17–17	155–172	115–129	28–30	pink or bright coral red, sometimes yellow

Due to the absence of a dark belly, *Hebiuscitrinoventer* sp. nov. can be distinguished from *H.annamensis* (Bourret, 1934), *H.chapaensis* (Bourret, 1934), *H.deschauenseei* (Taylor, 1934), *H.nigriventer* (Wall, 1925), *H.igneus* David, Vogel, Nguyen, Orlov, Pauwels, Teynié & Ziegler, 2021, and *H.youjiangensis* Yang, Xu, Wu, Gong, Huang & Huang, 2023 (vs. the dark belly present). Moreover, it can be distinguished from *H.deschauenseei* and *H.igneus* by having 17 DSR at midbody (vs. 19); from *H.chapaensis* and *H.nigriventer* by having 19 DSR on the anterior part of the body (vs. 17); from *H.annamensis* by having prefrontals 2 (vs. prefrontal single), VS 146–151 (vs. 158–172), IL 10–11 (vs. up to 10); and from *H.youjiangensis* by having TEMP 1+2 (vs. 1+1), PRO 2 (vs. 1), PO 3 (vs. 2), and dorsolateral series of irregular blotches (vs. a continuous stripe on dorsolateral).

By having 17 DSR at midbody, *Hebiuscitrinoventer* sp. nov. can be easily distinguished from the other 35 known species in the genus, which have 19 or 15 rows: *H.andreae* (Ziegler & Le Khac Quyet, 2006), *H.beddomei* (Günther, 1864), *H.bitaeniatus* (Wall, 1925), *H.boulengeri* (Gressitt, 1937), *H.celebicus* (Peters & Doris, 1878), *H.clerki* (Wall, 1925), *H.concelarus* (Malnate, 1963), *H.craspedogaster* (Boulenger, 1899), *H.flavifrons* (Boulenger, 1887), *H.inas* (Laidlaw, 1901), *H.ishigakiensis* (Malnate & Munsterman, 1960), *H.johannis* (Boulenger, 1908), *H.kerinciensis* (David & Das, 2003), *H.khasiensis* (Boulenger, 1890), *H.lacrima* Purkayastha & David, 2019, *H.leucomystax* (David, Bain, Quang Truong, Orlov, Vogel, Ngoc Thanh & Ziegler, 2007), *H.metusia* (Inger, Zhao, Shaffer & Wu, 1990), *H.modestus* (Günther, 1875), *H.miyajimae* (Maki, 1931), *H.nicobariensis* (Sclater, 1891), *H.octolineatus* (Boulenger, 1904), *H.optatus* (Hu & Zhao, 1966), *H.parallelus* (Boulenger, 1890), *H.petersii* (Boulenger, 1893), *H.popei* (Schmidt, 1925), *H.pryeri* (Boulenger, 1887), *H.sanguineus* (Smedley, 1932), *H.sangzhiensis* Zhou, Qi, Lu, Lyu & Li, 2019, *H.sarasinorum* (Boulenger, 1896), *H.septemlineatus* (Schmidt, 1925), *H.terrakarenorum* Hauser, Smits & David, 2022, *H.vibakari* (Boie, 1826), *H.viperinus* (Schenkel, 1901), *H.weixiensis* Hou, Yuan, Wei, Zhao, Liu, Wu, Shen, Chen, Guo & Che, 2021 and *H.yanbianensis* Liu, Zhong, Wang, Liu & Guo, 2018.

Compared with the other seven congeners that have 17 DSR at the midbody, *Hebiuscitrinoventer* sp. nov. can be distinguished from *H.arquus* (David & Vogel, 2010), *H.atemporalis* (Bourret, 1934), *H.frenatus* (Dunn, 1923), *H.sarwacensis* (Günther, 1872), *H.sauteri* (Boulenger, 1909), and *H.maximus* (Malnate, 1962) by the 19–17–17 DSR counts (vs. 17–17–15 in *H.arquus* and *H.frenatus*; and 17–17–17 in *H.atemporalis*, *H.sarwacensisH.sauteri* and *H.maximus*), from *H.groundwateri* (Smith, 1922) by the divided cloacal plate (vs. CP entire). Furthermore, this new species differs from *H.arquus* by having a single loreal (vs. the absence of loreal). It differs from *H.atemporalis*, *H.frenatus* and *H.sarwacensis* in terms of SL, namely (9 vs. 6 in *H.atemporalis*, 5–8 in *H.sauteri*, and 8 in *H.frenatus* and *H.sarwacensis*).

###### Description of holotype.

An adult female specimen with SVL 583 mm and incomplete tail (TAL 198+ mm). Body slightly stout and cylindrical; head flattened anteriorly, distinct from the neck, HL 26.1 mm, HW 15.6 mm. Nostril: lateral, round, piercing in the middle of the nasal. Eye large, ED 3.5 mm, pupil round.

***Body scalation***: DSR 19–17–17, feebly keeled, including the outermost DSR on both sides, not notched at the posterior extremity. VEN 144 (+2 preventrals); SC 80+, all paired; CP divided.

DSR reduction:

3+4→3 (79–80) (right)

19———————————17

3+4→3 (78–79) (left)

***Dentition***: Maxillary teeth 31, gradually enlarged, the last four slightly enlarged, without diastema between last four and anterior teeth.

***Head scalation***: Rostral pentagonal, wider than high, visible from above; nasal entire, subpentagonal, about twice as wide as high; internasals 2, trapezoidal, in broad contact with each other, narrowing anteriorly; prefrontals 2, pentagonal, wider than long, in contact with loreal; frontal narrow, pentagonal, longer than wide, shield-like, slightly concave in the middle on both sides; SPO 1 on each side, hexagonal, much longer than wide; LOR 1/1, subrectangular, wider than long; PRO 2/2, upper one larger than lower one; PO 3/3; SL 9/9, the first 2 in contact with nasal, the 2^nd^ to 4^th^ in contact with the loreal, 4^th^ to 6^th^ entering orbit, the 7^th^ and 8^th^ largest; TEMP 3/3, arranged in two rows (1+2), the anterior temporal long and trapezoidal; chin shields in 2 pairs, the posterior pair longer than anterior one and separated by several small scales; IL 11/11, first pair in contact behind the mental, 1^st^ to 5^th^ touching the first pair of chin shields, the 5^th^ and 7^th^ largest.

***Coloration of the fresh specimen***: Dorsal surface of the head is olive-brown and scattered with pale-brown vermiculate stripes or irregular blotches. A pale, irregular yellow-ochre oblique streak is directed upward on both sides of the head, extending from the temporal region to the nape. The upper half of the 1^st^ to 8^th^SL is olive-brown, the lower half is pale brown, and the 9^th^ is completely olive-brown. Ventral surface of head creamy yellow, the edges of partial scales have irregular black-gray patches.

Body olive-brown, darker on the top than on the sides. A faint, yellow-ochre or rusted dorsolateral stripe extends from the neck to the end of the tail, on the upper part of the 5^th^ to the lower part of the 7^th^ scale rows in the anterior part of the body, and the upper part of the 4^th^ to 6^th^ in the middle and posterior parts of the body, accompanied by a series of conspicuous, pale orange or ochre yellow irregular blotches, about two scales in diameter. The orange or ochre yellow blotches are not symmetrically distributed on both sides of the body but are arranged in a staggered manner, that is, the blotch on the left side of the body corresponds to the area between the two blotches on the right side of the body, and vice versa. Above and below the orange blotch, there is a slightly smaller, dull blackish-brown irregular blotch. The blackish-brown blotches above are arranged in a staggered pattern in the middle of the body, forming a checkered pattern with the background color.

Ventral anterior pale orange, darker toward the rear, and scattered with a few small black spots. The outermost edge of the ventral is black. Outer one-sixth of the ventrals with subrectangular black blotches on each side, producing an irregular, continuous ventrolateral stripe, which merges with the dark ventral edge in the posterior part of the body. The ventral surface of the tail is uniform pale orange with black-brown edge; a thin, brown-black stripe extends on the ventral part of the tail, formed by the inner margins of the SC, extending from the 1^st^SC to the end of the tail.

***Coloration in preservation***: In preservation, the background color of the dorsal body changed to brownish-black, and the checkered pattern on dorsal surface has disappeared or faded. An indistinct pale-brown dorsolateral stripe extends from the neck to the end of the tail and is accompanied by a series of ochre yellow, irregular blotches. Head brownish-black, upper half of the 1^st^ to 8^th^ supralabials brownish-black, lower half gray white, the 9^th^ completely brownish-black. The infralabials mainly black-gray, the left half of the 5^th^ to 11^th^ pale gray, and the right half very dark gray. Ventral surface of the head cream, the edges of the partial scales had irregular very dark gray patches. The ventral surface of the body cream anteriorly, darker toward the rear, and the posterior part is light creamy yellow. In addition, the rest of the color pattern is similar to that seen in life.

###### Variation.

The paratype has a similar coloration in preservation as the holotype, but the subcaudals are almost completely black, with only the inner margins being creamy yellow. In scalation features, there is the following variation: the paratype has fewer infralabials (10 vs. 11) and more ventrals (151 vs. 146). The measurements and scalation features of the series (*N* = 2) are listed in Table 4.

**Table 4.** Main morphological characters of *Hebiuscitrinoventer* sp. nov. Abbreviations as per Material and methods.

Voucher Number	ANU20230016	QHU2024005
	Holotype	Paratype
Sex	Adult female	Subadult male
SVL	583	267
TAL	198+	134
TL	781+	401
TAL/TL	–	0.334
HW	15.6	6.7
HL	26.1	12.6
ED	3.5	1.9
MT	31	–
SL	9/9	9/9
SL-Eye	4^th^–6^th^	4^th^–6^th^
IL	11/11	10/10
Chin	2	2
IL-1^st^ Chin	1^st^–5^th^	1^st^–5^th^
LOR	1	1
PRO	2	2
PO	3	3
TEMP	1+2	1+2
DSR	19–17–17	19–17–17
VS	146	151
SC	80+	113
CP	2	2

###### Distribution and habitat.

**Figure 7.**
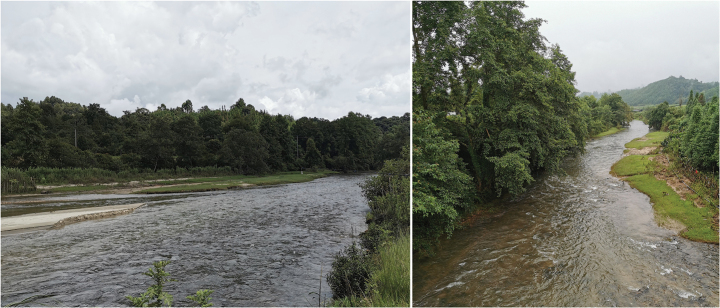
Habitat of *Hebiuscitrinoventer* sp. nov., Tongbiguan Town, Yingjiang County, Yunnan Province. Photos by Kai-Chen Ouyang.

*Hebiuscitrinoventer* sp. nov. is currently only known from Yingjiang County, Dehong Dai and Jingpo Autonomous Prefecture, Yunnan Province, China: Tongbiguan Town (1300 m a.s.l.) (Fig. 7). The holotype (ANU20230016) was found drowned in a fish-catching cage placed by local residents in a wide stream at approximately 7:00 am, and we presume that it may have fallen into the trap the preceding night while attempting to catch fish in the cage. The paratype (QHU2024005) was found road-killed on the side of the road next to a stream after a light rain between 21:00 and 22:00. Both specimens were found in a well-preserved monsoon forest habitat.

## ﻿Discussion

In this study, we combined morphological and molecular analyses of specimens in the genus *Hebius* to provide robust evidence for the identification of new species. Based on molecular phylogenetic analysis, we found that the sequence KJ685679 (voucher number: GP 1618) from Myanmar clustered together with *H.taronensis* and had a very low *p*-distance of approximately 0.3% in Cyt *b*. However, it was referred to as *Hebius* sp. in Guo et al. (2014) but tentatively as *H.venningi* in David et al. (2021). Because the specimen was not examined, we conservatively assigned it to *H.taronensis*. Additionally, the uncorrected *p*-distance between specimen AMNH 147155 from Vietnam and *H.optatus* was 9.8%, which clearly reached the inter-species level; therefore, we proposed the specimen as H.cf.optatus. Likewise, the phylogenetic structure showed that the specimens (GP 1940 and AUP-00062), which were once considered *Hebiusbitaeniatus*, clustered in different clades: *H.craspedogaster*, *H.johannis*, *H.metusia* and *H.octolineatus* were clustered together with low support and formed a sister group with specimen GP 1940; the specimen AUP-00062 was clustered with *H.septemlineatus* with high support (SH 93 / UFB 99), and the uncorrected *p*-distance was 4.2%. However, due to the lack of morphological and molecular data on the topotype, the classification status of these two specimens requires further study.

The new species, *Hebiuscitrinoventer* sp. nov., has some morphological features common to *H.venningi* and *H.taronensis*, but can still be distinguished from them in the following characters: dorsal scale rows, number of temporal and ventral scales, and venter color pattern. Molecular phylogenetic analysis also separated the new species *H.citrinoventer* sp. nov. and provided strong support for its placement as a sister taxon. Moreover, the new species also possessed a considerable level of genetic divergence from 9.7–10.0% for *H.venningi* and 10.9–11.2% for *H.taronensis* in Cyt *b*. In addition, the new species is geographically isolated by the Hengduan Mountains, which plays an important role as a geographical barrier in speciation.

The genus *Hebius* is a highly diverse group distributed throughout eastern and southern Asia. Owing to the semi-aquatic habitats of this genus, specimen collection is relatively difficult, and the population and distribution data for many species is insufficient, which poses obstacles to conservation. Yingjiang County, where the new species was found, lies in the southwest Yunnan Province and is one of the most biodiverse regions in China. Although the discovery site of this new species is legally protected, the holotype derived from the fish trap and the road-killed paratype clearly indicates that this species is still influenced by human activities. Further surveys and evaluation of the population of the new species should be performed, and further consideration should be given to incorporating it in the local protected animal lists for protection.

In China, most species of *Hebius* are known from Yunnan Province, and the identification of *Hebiuscitrinoventer* sp. nov. brings the total number of *Hebius* species in China to 27, of which 20 are reported in Yunnan Province. This result further illustrates that reptile diversity in Yunnan is still underestimated. Therefore, more specific surveys may help to better understand the biodiversity in southwest China.

## ﻿Conclusion

Here, we describe a new species of the genus *Hebius*, *Hebiuscitrinoventer* sp. nov., based on two specimens collected from Yingjiang County, Dehong Dai and Jingpo Autonomous Prefecture, Yunnan Province, China. The discovery of this new species has brought the total number of known species in the genus *Hebius* to 52. Currently, *Hebiuscitrinoventer* sp. nov. is only known to be distributed in southwest Yunnan Province, China. Since Yingjiang County is close to the borders of Myanmar, this species also probably occurs in the adjacent area of this country. However, the detailed distribution range, population size, and feeding habits of the new species have not yet been elucidated, and further research and evaluation of the population of the new species should be conducted.

## Supplementary Material

XML Treatment for Hebius citrinoventer

## ﻿Additional information

### Author ORCIDs

Yu-Hao Xu https://orcid.org/0000-0001-6094-6680

Dian-Cheng Yang https://orcid.org/0000-0001-6390-5251

Yan-An Gong https://orcid.org/0000-0002-8155-7024

Kai-Chen Ouyang https://orcid.org/0009-0003-6876-4699

Song Huang https://orcid.org/0000-0001-6786-8523

Li-Fang Peng https://orcid.org/0000-0001-9325-7048

### Data availability

All of the data that support the findings of this study are available in the main text.
